# Using a shortened uncuffed endotracheal tube as a nasopharyngeal airway: a useful adjunct during fiberoptic intubation training among anesthesia residents

**DOI:** 10.4314/ahs.v23i3.67

**Published:** 2023-09

**Authors:** Hosni A Salem, Ayman Aly Rayan, Usama Abotaleb, Essam shafiq M Abdel-wahap, Ismail A Elzoughari, Mohammed A Taha Alafifi, Walid Kamal Abdelbasset, Ahmed M Abodonya

**Affiliations:** 1 Department of Anesthesia and Intensive Care, Faculty of Medicine, Al-Azhar University, Cairo, Egypt; 2 Department of Anesthesia and Intensive Care, Faculty of Medicine, Menoufia University, Menoufia, Egypt; 3 Department of Physiotherapy, College of Health Sciences, University of Sharjah, Sharjah 27272, United Arab Emirates; 4 Department of Health and Rehabilitation Sciences, College of Applied Medical Sciences, Prince Sattam Bin Abdulaziz University, Al-Kharj 11942, Saudi Arabia; 5 Department of Surgery, College of Medicine, Prince Sattam Bin Abdulaziz University, Al-Kharj 11942, Saudi Arabia

**Keywords:** Nasopharyngeal airway, Endotracheal tube, Training of FOI

## Abstract

**Background:**

Fiberoptic intubation (FOI) is considered a beneficial modality used to intubate life-threatening airway patients. This study aims at assessing the effectiveness of shortened uncuffed endotracheal tube as a nasopharyngeal airway during FOI.

**Methods:**

Between January 2019 and March 2021, this prospective randomized controlled trial has enrolled 62 adult patients (56 males and 6 females) with normal airways scheduled for elective oral FOI classified American Society of Anesthesiologists (ASA I-III), their age ranged 20-60 years. The patients were randomized into two equal groups (31 per each); in group I, FOI was carried using lingual traction, and in group II, FOI was carried out with lingual traction plus a shortened uncuffed endotracheal tube as a modified nasopharyngeal airway to maintain oxygenation. The time taken to successful tracheal intubation and other technical parameters have been measured. The heart rate (HR), mean arterial pressure (MAP), oxygen saturation (SpO_2_), end-tidal carbon dioxide (EtCO_2_), and any associated complications have been measured.

**Results:**

During insertion of the scope, the SpO2 was significantly decreased in group I (92.55 ± 7.94) compared to group II (97.42 ± 6.34), p=0.009. The heart rate, MAP, and EtCO_2_ were found to be insignificantly different in both groups (p>0.05). The time needed for intubation in group I (2.78±0.98 min) was prolonged compared with group II (1.95±1.02 min) p =0.002. The number of attempts was comparable in both groups, while the number of successful intubations from the 1st attempt was 12 (39%) compared to 18 (58%) in groups I and II respectively, p=0.36. The overall success rate by juniors was 71% in group I compared to 84% in group II, p=0.66 with a lower incidence of using rescue oxygen and other facilitating maneuvers.

**Conclusions:**

The modified nasopharyngeal airway is a useful modality to facilitate oral FOI by anesthesia resident trainees.

## Introduction

Tracheal intubation is usually needed in different surgeries. Number of cases exhibits difficult airways with a prevalence rate of 0.3-13% 1, 2. Among 250 cases, one is having trouble mask ventilation and laryngoscopy that interfere appropriate intubation and oxygenation 3, 4. In addition, inappropriate control of difficult intubation may lead to hypoxic brain damage resulting in high rate of morbidity and mortality 5,6. Old techniques like blind nasotracheal intubation, retrograde intubation, and cricothyrotomy have been replaced by bougie, supraglottic devices, Bullard laryngoscope, video optical intubation stylet, McGrath laryngoscope, Glidescope, Air Track as well as fiberoptic intubation 7. Fiberoptic intubation is the gold standard and has become an essential technique in the management of difficult airway 8.

The FOI is ideally suited in such patients with risks of inadequate ventilation and oxygenation, loss of upper airway patency, and failed intubation. This FOI technique also safeguards against the risk of the cannot intubate/cannot ventilate scenario 9. To facilitate FOI, various conduits have been used in the past like the intubating oral airways, the intubating laryngeal mask airway (LMA), and the nasopharyngeal air way (NPA)^10^.

The success in the use of the intubating fiberscope mainly depends on the experience and skill of the anesthesiologist. Various methods of training were evaluated. They include didactic lectures, workshops with hands-on training on the patients under supervision, use of airway training manikins, simulators, virtual reality trainers and cadavers 11. In anesthesia residents' training program, we used the shortened uncuffed tube as a nasopharyngeal airway connected to the anesthesia machine to maintain oxygenation and to assess ventilation during oral FOI by end-tidal carbon dioxide (EtCO2).

To the best of our knowledge, no prior studies have been designed to assess the effectiveness of shortened uncuffed endotracheal tube during FOI. Therefore, our current study is the first to assess the efficacy of modified nasopharyngeal airway during oral FOI theorizing that the learning of FOI by the anesthesia resident trainee is easier and safe.

## Materials and Methods

### Study design, Settings, and Ethics

This randomized controlled clinical trial has been conducted in King Khalid Hospital after approved ethically by the institutional research ethics committee in health and science disciplines at Prince Sattam bin Abdulaziz University (REC-HSD-67-2021). The study was registered with ClinicalTrials.gov Identifier: NCT04852263. After explaining the benefits and predicted complications of the procedures, a written consent form has been obtained from each patient before starting the study.

Third-year anesthesiology residents were recruited as subjects. They received a manual that contained step-by-step instructions on how to perform an FOI. After their respective training sessions, six subjects were enrolled in the study

### Participants

Between January 2019 and March 2021, this prospective randomized controlled trial has enrolled 62 adult patients (56 males and 6 females) with normal airways were scheduled for elective oral FOI classified American Society of Anesthesiologists (ASA I-III) with Mallampati score I or II, their age ranged 20-60 years. The patients were randomized into two equal groups (31 per each); in group I, FOI was carried using lingual traction (control group). In group II, FOI was carried out using lingual traction plus a shortened uncuffed endotracheal tube used as a modified nasopharyngeal airway (m-NPA group). Patients who have refused to undergo the procedure, heart, or lung disease, obese (BMI > 30), pregnancy, and presence of any contraindication to nasal intubation like head trauma, a nasal mass, and deviated nasal septum have been excluded from the study. Data collections are recorded by the anesthesia resident that not included in the procedure.

### Sample Size Estimation

Using G*Power Software (Version 3.1.9.2, Universität Düsseldorf, Germany), the sample size has been estimated. According to our previous preliminary study that included 6 patients, 3 per each, the effect size of SpO2 was |5.21|. With two-tailed t-test, α= 0.05, β= 0.2, and 80% power, the required sample size was 54, 27 per each group. Therefore, we recruited 62 patients to nullify the 20% withdrawal rate.

### Randomization and Blindness

To avoid bias, the study participants have been randomized into two equal groups, 31 per each group using a random number generating table. The allocation has been done before initiating the study program by a blinded examiner who was uninformed of the group intervention.

### Intervention

Patients have been optimized for surgery after 8 hrs fasting. Each resident has been trained about the basics of fiberoptic bronchoscopy many times on airway manikins before participating in the study. While the patients in the preoperative holding area, they have been premedicated with glycopyrrolate 0.2 mg intravenously 30 min prior to the procedure as an antisialagogue. At the same time, patients have been treated with xylometazoline 0.1% nasal drops in both nostrils followed by lignocaine jelly 2%.

**Figure 1:**
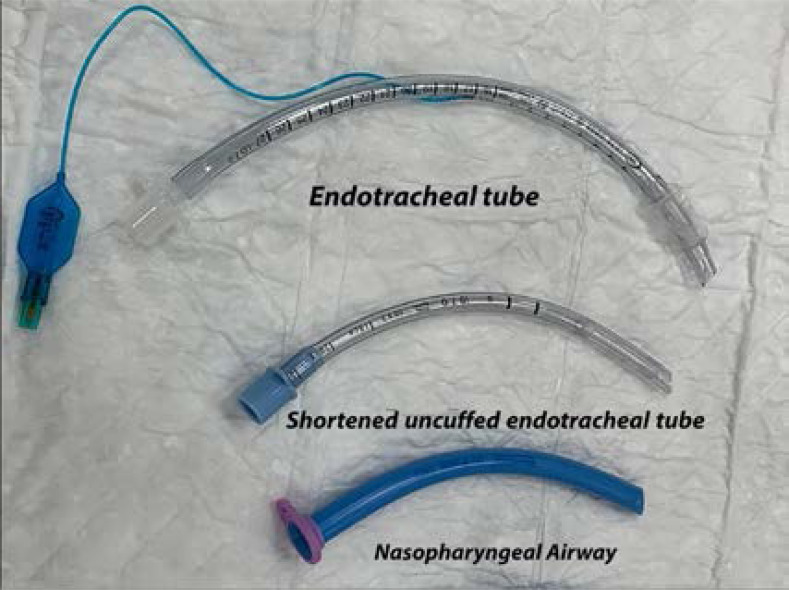
Shortened Uncuffed Endotracheal tube

In the operating room, patients have been connected to standard monitors including pulse oximetry, ECG, non-invasive blood pressure, and temperature monitoring. Then, midazolam 2 mg IV, and remifentanil 1µg/kg intravenously over 2-3 min have been slowly injected 3 min prior to the procedure then maintained with 0.1-0.5 mcg/kg/min for all patients included in the study. In group II (m-NPA group), when the patient has been sedated, a well-lubricated warmed uncuffed tube (Portex®) 7.0 mm for male and 6.5 mm for female) has been inserted nasally after measuring from the tip of the nose to the tragus of the ear and then connected to anesthesia machine, Drager Primus manual mode with side-stream capnography through 15 mm tube connector (Figure 1). After a period of 2-3 min of preoxygenation, the patient has been anesthetized with total intravenous anesthesia (TIVA) using propofol infusion co-administered with remifentanil infusion. The anesthesia resident trainee has been instructed to use a fiberoptic bronchoscope (Karl Storz® Intubation fiberscope 11301 BN1) to pass it through a cuffed endotracheal tube (ETT). Lingual traction has been used in both groups by grasping the tongue with 4x4cm gauze and gently pulling the tongue out until resistance is met. Visualizing the vocal cords and the intubating dose of muscle relaxant (rocuronium bromide 1 mg/kg) injected then the fiberoptic pass through the glottic aperture and then pass the ETT over the fiberoptic bronchoscope. The correct ETT placement has been observed by visualization of the carina and tracheal rings and bilateral equal air entry and confirmed by the presence of EtCO_2_ waveform, the endoscope has been removed and positive pressure ventilation has been established.

The same scenario has been done by the resident trainee in group I but without using the m-NPA. Alternatively, we used side-stream capnograph in collaboration with simultaneous oxygen administration via a nasal prong (Omniline; Adult Oral-Nasal Sampling Line, O_2_, OM, Length 7″ (2.0m). If tracheal intubation has failed within 180 sec or SpO_2_ decreased below 94%, the fiberoptic bronchoscope has been removed, and an experienced anesthetic has performed the FOI after 2 min 100% oxygen manually ventilated. The procedure has been supervised by professional experienced anesthesiologists (study investigator) providing guidance and direction to the resident.

### Assessment

The primary outcome measure was SpO_2_ while the secondary outcome measures have included the intubation time (stared from the insertion of the fiberscope until the appearance of EtCO_2_ waveform), number of attempts, hemodynamics, and respiratory changes (HR, MAP, and EtCO_2_). All measures have been recorded at 3 points; at the baseline before induction of anesthesia, during insertion of the scope (two minutes after induction of anesthesia), and after FOI (two minutes after the appearance of the first end-tidal CO_2_ waveform). If necessary, facilitating techniques like jaw thrusts were used. Any adverse events were also recorded as nasal bleeding, dental trauma, desaturation, bradycardia sore throat or PONV.

### Statistical analysis

The statistical analysis has been conducted using a Statistical Package for the Social Sciences - SPSS Statistics for Windows (SPSS Inc., Chicago, Illinois, USA) version 17. Parametric variables have been presented as mean ± standard deviations, while non-parametric variables have been presented as numbers and percentages. Student's t-test has been used to analyse the changes in parametric variables, while Mann Whitney U and Chi-square tests have been used to assess the changes in non-parametric variables. The data have been checked for normality using a Shapiro-Wilk test. A p-value of less than 0.05 has been set as significant.

## Results

The demographic data of patient groups (age, sex, BMI, ASA levels, and Mallampati score) have been comparable and the differences have been non-significant (Table 1).

**Table 1:** Age, sex, BMI, ASA and Mallampati scores

Parameter	Group I Mean ± SD No=31	Group II Mean ± SD No=31	P value
Age	48.00 ± 12.38	48.42 ± 12.03	0.893^*^
Males/Females	29/2	27/4	0.671^#^
BMI	24.54±3.12	25.6±2.33	0.135^*^
ASA score (I: II: III)	3:8:20	4:8:19	0.919^¶^
Mallampati class (1:2)	10:21	13:18	0.599^¶^

* t-test

# Fisher's exact

¶ chi-square tests

BMI: Body mass index, ASA: American Society of Anesthesiologists

**Figure 2:**
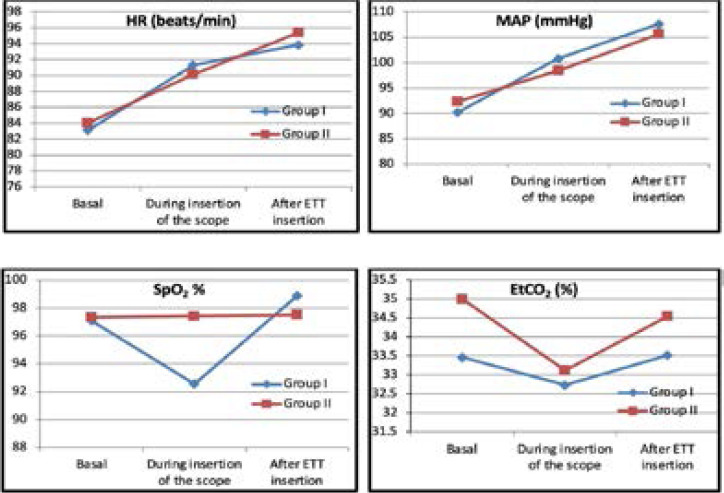
HR, MAP, SpO2 and EtCo2 in the two groups

As detailed in Table 2, the HR, MAP, and EtCO2 have been compared in both groups. A statistically significant decrease of SpO2 has been observed in group I during FOI (92.55±7.94 %) compared with group II (97.42±6.34 %) with p=0.009 (Figure 2). The time taken for intubation has been significantly longer in group I compared with group II (2.78±0.98 vs 1.95±1.02 min) with p=0.002. The number of attempts has been also compared in both groups, while the number of successful intubations from the 1st attempt has been 12 compared to 18 in groups I and II respectively. The overall success rate by juniors has been 71% and 84% in groups I and II respectively. Rescue oxygen has been used in 9 cases in group I compared with 5 cases in group II. Facilitating jaw thrusts has been used in 5 cases in group I and 2 cases in group II with no significant differences between them (Table 3).

**Table 2:** Comparative HR, MAP, SpO_2_ and EtCO_2_ among studied groups

Time interval	Group I Mean ± SD No=31	Group II Mean ± SD No=31	*t*-value	*p*-value
**HR (beats/min)**				
Baseline before induction of anesthesia	83.12 ± 9.12	84.0 ± 9.26	0.377	0.707
During insertion of the scope*	91.29 ± 10.66	90.11 ± 12.35	0.403	0.688
After ETT insertion	93.84 ± 8.66	95.42 ± 10.93	0.631	0.530
**MAP (mmHg)**				
Baseline before induction of anesthesia	90.18 ± 8.74	92.29 ± 8.42	0.968	0.337
During insertion of the scope	100.80 ± 9.29	98.43 ± 8.35	1.056	0.295
After ETT insertion	107.65 ± 8.34	105.64 ± 11.49	0.788	0.434
**SpO_2_**				
Baseline before induction of anesthesia	97.10 ± 8.64	97.33 ± 7.84	0.110	0.913
During insertion of the scope	92.55 ± 7.94	97.42 ± 6.34	2.669	0.009
After ETT insertion	98.88 ± 8.04	97.49 ± 6.08	0.768	0.445
**EtCO**_**2**_ **(%)**				
Baseline before induction of anesthesia	33.46 ± 4.24	35.00 ± 3.22	1.610	0.112
During insertion of the scope	32.73 ± 3.82	33.12 ± 3.67	0.410	0.683
After ETT insertion	33.52 ± 3.19	34.55 ± 4.02	1.117	0.268

Two minutes after induction of anesthesia, HR: heart rate, MAP: Mean arterial blood pressure, SpO_2_: Mean oxygen saturation pressure, EtCO_2_: Endtidal tension of carbon dioxide, ETT: Endotracheal tube

**Table 3:** Technical parameters and intubating conditions among studied groups

Time interval	Group I Mean ± SD No=31	Group II Mean ± SD No=31	t-value	p-value
Intubation time (min.)	2.78±0.98	1.95±1.02	3.267	0.002
No. of attempts	1.38±57	1.2±41	1.34	0.18
No. of intubation from the 1^st^ attempt	12	18	0.833	0.361
Overall success rate by juniors (No.)	22 (71%)	26 (84%)	0.187	0.665
Use of rescue drug (atropine)	0	0		
Use of rescue oxygen	9	5	0.643	0.422
Use of facilitating maneuver (Jaw thrusts)	5	2	0.571	0.449

No gross adverse events have been recorded in studied cases, only two cases complaining of mild epistaxis in group II.

## Discussion

The current study was designed to assess the efficacy of modified nasopharyngeal airway during oral FOI theorizing that the learning of FOI by the anesthesia resident trainee is easier and safe. The study results confirmed our hypothesis in the short duration and maintaining SpO_2_ during FOI.

Fiberoptic endoscope is the most valuable single tool available for the anesthetist to manage the difficult airway and is considered as the gold standard in airway management 7. To facilitate FOI, various conduits have been used in the past like the intubating oral airways, the intubating laryngeal mask airway (LMA), and NPA 12. Our study was undertaken to compare the ease of training of oral FOI using shortened uncuffed endotracheal tube as a modified NPA connected to an anesthesia machine keeping oxygenation and assessing the ventilation to the conventional FOI without m-NPA. We found that a statistically significant decrease of SpO_2_ was observed in group I during FOI compared with group II, this is due to the use of oxygen during the procedure. The anesthesia resident intubates the patient at ease without fearing hypoxia in one shot without interruption for oxygenation, so the time taken for intubation was significantly longer in group I compared with group II. And the number of successful intubations from the 1st attempt was decreased in the m-NPA group and the overall success rate increased in the m-NPA group due to the anesthesia resident manipulating in a good situation.

Compared to our study, various conduits have been used to minimize the number of attempts in fiberoptic intubation. Patil et al. 7 compared the success rate of nasal FOI with & without NPA. They found a success rate of 100% in both groups. The mean number of attempts required for successful intubation was less in FOI with the NPA group (1.31 ± 0.47) as compared to FOI without the NPA group (1.97±0.86) and it was statistically significant. But the mean intubation time in FOI with the NPA group was 3.52 ± 1.25 min, and that in FOI without NPA group was 2.19 ± 0.86 min which was statistically significant (p < 0.05). A study by Muthukumar et al., 13 designed to evaluate the simple predetermined length insertion technique (SPLIT) during oral FOI with conventional FOI. They found the time was significantly less in SPLIT as compared to the conventional technique. The time taken by SPLIT was comparable between residents and consultants (P = 0.09), whereas it was significantly more among residents in the conventional technique. They concluded that SPLIT significantly lessened the time to visualize the glottis than a conventional technique for FOI. Patil et al., 7 reported that the rate of desaturation, hypotension, and bradycardia was higher in the modified NPA group compared to the conventional group (p > 0.05). These findings explained that the modified NPA group used in this study as a conduit facilitating the passage of FOI not for oxygenation, and the time of FOI therefore was lengthened in Patil et al study due to the insertion of m-NPA as sequence of the technique. However, in our study we used m-NPA as a source of oxygen and the insertion time of m-NPA was excluded that didn't affect our measurements (the time of FOI maneuver). Mohammad Zadeh et al., 14 used the endotracheal tube as a conduit and concluded that a significant reduction in incubation time was observed with conduit (1.27±0.41 min) than the conventional method (2.93±0.56 min). Adachi et al., 15 reported that insertion of the endotracheal tube is the most invasive stimulus causing a hemodynamic response in the form of an increase in heart rate and blood pressure. In our study, proper doses of premedication and remifentanil infusion maintained the hemodynamic stability throughout the procedure.

No gross adverse events were recorded in our study. Only two cases complaining from mild epistaxis in m-NPA group. Compared to our study, Patil et al., 7 reported that the rate of epistaxis, trauma and mucosal ulceration were significantly higher in FOI with NPA group as compared to FOI without NPA group (p <0.05), but no patient required any rescue treatment. In Meena et al., 10 study, nasal bleeding was seen in 10% of the patients in the spirally split rubber nasopharyngeal group, as compared to 52.6% in the ETT group.

Some strengths have been detected in the study. Firstly, providing patient safety and supporting anesthesia resident trainees to avoid stress and to improve learning outcomes. Secondly, modified NPA maintained SpO_2_ and hemodynamics, and shortened the time taken for intubation. Lastly, this study is the first such study that assessed the efficacy of modified NPA during oral FOI.

However, some limitations have been demonstrated. The study has included a small sample size of 62 despite power and size have been estimated that may affect the generalization of the study results. Further, it is better to create a rubber NPA connected to 15 mm connectors to anesthesia machine to replace the shortened endo-tracheal tube to minimize nasal trauma during FOI. Future studies should include large sample size and use a rubber NPA connected to anesthesia machine.

## Conclusion

Fiberoptic intubation requires a lot of practice and proficiency. Based on the present study and the results obtained, it can be concluded that modified NPA seems to be a reliable and safe technique in training of anesthesia resident trainees on oral fiberoptic intubation.
